# Study of the Volatile Constituents in Radix Flemingiae Macrophyllae and a Substitute by Gas Chromatography-Mass Spectrometry and Chemometric Methods

**DOI:** 10.3390/molecules171214111

**Published:** 2012-11-28

**Authors:** Shen-Yu Cheng, Yan Xie, Xiao-Liang Feng, Lan-Fang Huang

**Affiliations:** School of Chemical and Material Engineering, Quzhou College, Quzhou 324000, China

**Keywords:** Radix Flemingiae Macrophyllae, chemometrics, gas chromatography-mass spectrometry, subwindow factor analysis, spectral correlative chromatography, correlative constituent

## Abstract

A combined approach of subwindow factor analysis and spectral correlative chromatography was used to analyze the volatile components in Radix Flemingiae Macrophyllae and Flemingiae Latifolia Benth, one of its substitutes. After extraction by a water distillation method, the volatile components in Radix Flemingiae Macrophyllae and Flemingiae Latifolia Benth were detected by GC-MS. Then the qualitative and quantitative analysis of the volatile components in Radix Flemingiae Macrophyllae was completed with the help of subwindow factor analysis resolving two-dimensional original data into mass spectra and chromatograms. Sixty five of 82 separated constituents in the total ion chromatogram of the volatile components in Radix Flemingiae Macrophyllae were identified and quantified, accounting for about 88.79% of the total content. Then, spectral correlative chromatography was used to extract correlative constituents in Flemingiae Latifolia Benth. Fifty one correlative components were recognized in essential oil of Flemingiae Latifolia Benth. The result proves the combined approach is powerful in the analysis of complex herbal samples. The developed method can be used to compare the sameness and differences of Radix Flemingiae Macrophyllae and its substitutes and it can also be used for quality control of Radix Flemingiae Macrophyllae.

## 1. Introduction

Radix Flemingiae Macrophyllae, belongs to the leguminous plant family. As a traditional Chinese medicine (TCM) listed in the Chinese Pharmacopoeia, it has been widely used to cure rheumatism, lumbar muscle strains, myasthenia of limbs, injuries from falls, swelling, sore throats, *etc*. [1,2,3]. Although Radix Flemingiae Macrophyllae contains tens or even hundreds of compounds, only a limited number of compounds, such as flavonoids and the volatile constituents, might be responsible for the pharmaceutical or toxic effects [4,5,6]. For example, the volatile constituents are known to exhibit pharmacological and biological activity, and are used for the therapy of various liver and gall bladder diseases [7]. In recent years, because Radix Flemingiae Macrophyllae has been widely harvested for a long time, its supplies have become scarce and many fakes or substitutes, such as Radix Flemingiae Latifolia Benth, appeared on the market. To ensure the reliability and repeatability of pharmacological and clinical research and understand their bioactivities and possible side effects of active compounds, it is necessary to study all of the phytochemical constituents of botanical extracts and develop a method for quality control of Radix Flemingiae Macrophyllae. 

Analysis of the volatile compounds in Radix Flemingiae Macrophyllae is usually performed with gas chromatography (GC) and gas chromatography–mass spectrometry (GC-MS) [8,9]. Because the composition of Radix Flemingiae Macrophyllae is very complicated and the concentrations of many important volatile components in Radix Flemingiae Macrophyllae are very low, suitable sample-preparation methods are necessary before detection by GC-MS, such as steam distillation [8,9]. However, although sample preparation methods are used for the complicated Radix Flemingiae Macrophyllae samples, it is still difficult to achieve a complete separation unless rigorous conditions are imposed on the chromatographic separation process. In these reports, the qualitative and quantitative analysis of the volatile components determined by gas chromatography–mass spectrometry (GC-MS) is based on retention index of gas chromatography and mass spectra. However, it is difficult to assess the purity of chromatographic peaks by general GC and the peaks inspected as one component may be mixtures of several components, so the results obtained by the methods mentioned above would be questionable. Therefore, development of a simple and reliable method for the determination of the volatile constituents in Radix Flemingiae Macrophyllae and its substitutes is necessary. Fortunately, many associated chemometric methods [10,11,12,13,14,15,16] have been developed to provide more information for chemical analysis both in chromatographic separation and in spectral identification, which makes it possible to interpret these complex systems [17,18].

In this work, combined chemometrics methods, subwindow factor analysis (SFA) [14] and spectral correlative chromatography (SCC) [15], are used for analysis of the volatile constituents in Radix Flemingiae Macrophyllae and Radix Flemingiae Latifolia Benth., one of its substitutes. Then, a simple, reliable and reproducible procedure to reveal the sameness and differences between Radix Flemingiae Macrophyllae and its substitutes is developed. After extraction by the water distillation method, the volatile components in Radix Flemingiae Macrophyllae and Radix Flemingiae Latifolia Benth. were detected by GC-MS under appropriate conditions. The qualitative and quantitative analysis of volatile components in Radix Flemingiae Macrophyllae was completed when subwindow factor analysis was used as an auxiliary means. Then, spectral correlative chromatography (SCC) was used to extract the correlative constituent from Radix Flemingiae Latifolia Benth. The obtained results proved the power of the developed approach for the analysis of complex samples.

## 2. Results and Discussion

### 2.1. Resolution of Overlapped Peaks with SFA

**Figure 1:**
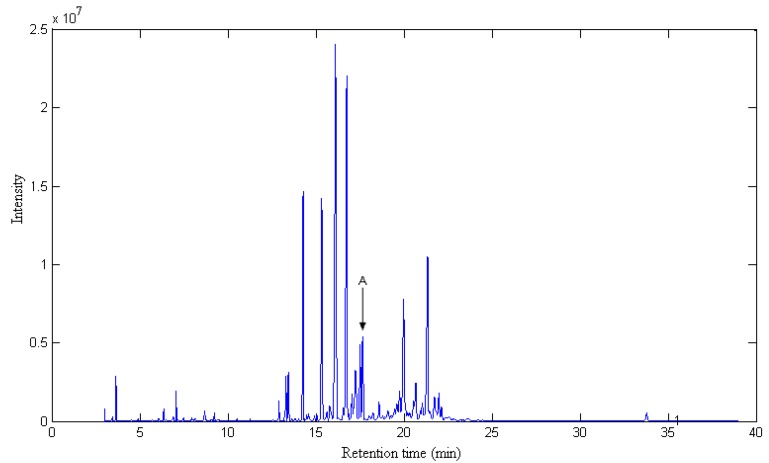
The total ion chromatogram (TIC) of the volatile components in Radix Flemingiae Macrophyllae.

The total ion chromatogram (TIC) of the volatile components in Radix Flemingiae Macrophyllae is shown in Figure 1. Apparently, it is indeed a very complicated system, and the majority of the peaks are grouped with retention times within the 12.50–25.00 min. range. If we searched in the NIST mass database directly without further data processing, incorrect results or impossible identification would result obtained. The main reason is that although the chromatographic separation conditions were optimized, some of the eluting components overlap with one another, as indicated by the fact that different mass spectra were obtained at different positions in the peaks when we searched in the NIST database, and the furthermore, the concentrations of many volatile components are very low. In order to obtain reliable qualitative and qualitative results of the volatile components in Radix Flemingiae Macrophyllae, it was necessary for the overlapped peaks and the components with low content to be resolved into pure spectra and chromatograms using combined chemometrics resolution methods. Subwindow factor analysis (SFA) [14], a common used chemometric method for solving these problem, was used for this purpose. 

**Figure 2:**
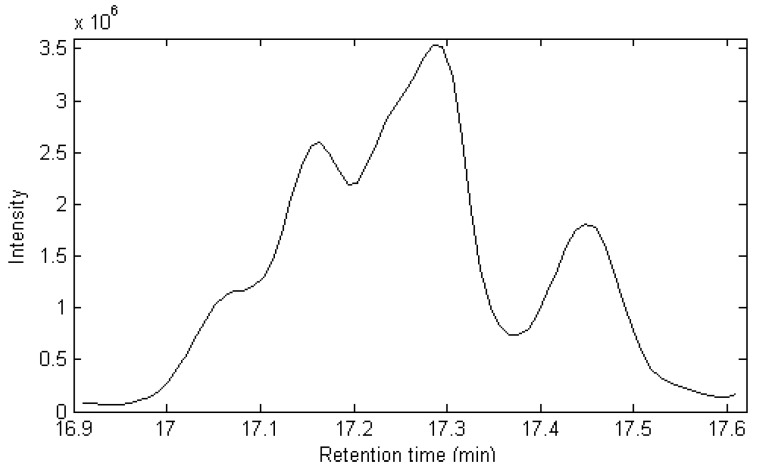
The total ion Chromatogram(TIC) of the peak cluster A.

The TIC of peak cluster A (16.90–17.60 min) in Figure 1 is taken as an example to demonstrate the whole procedure of this approach. Figure 2 shows the total ion chromatogram (TIC) of this peak cluster, which seems to be a three component overlapped peak, but the diverse spectra at different parts of the cluster indicates there are actually more than three components in the cluster. A satisfactory result can’t be obtained by the usual direct search in the mass spectrum library. Of course, the quantitative analysis of this cluster can’t be completed because the area of each component can’t be determined. Thus, sub-window factor analysis is used to extract the pure spectra and pure chromatogram. 

**Figure 3:**
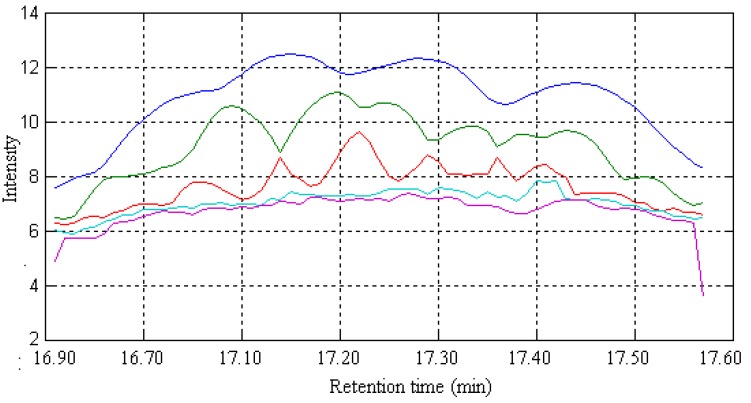
The evolving eigenvalues of peak A obtained using FSMWEFA with a window size of 5.

**Figure 4:**
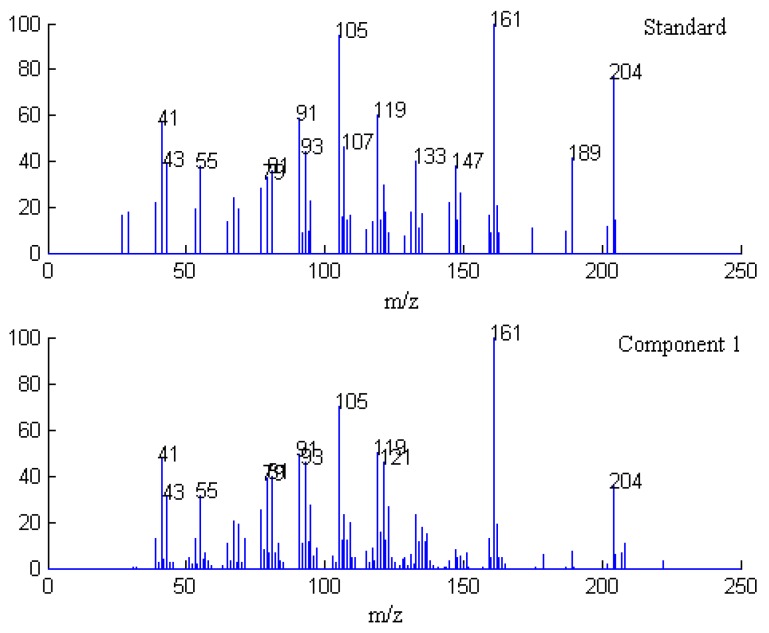
Resolved mass spectrum of component 1 by SFA and standard mass spectrum of β-guaiene.

After background and baseline shift in the raw dimensional data are preprocessed by the appropriate method [13], the elution sequences of individual components (rank map of peak A) can be estimated by FSMWEFA [16], which is shown in Figure 3. It can be seen there are seven components in this peak cluster according to the rank estimation method, which are marked as components 1–7 according to elution sequence. By analyzing the correlation of two subwindows, the pure spectrum of each component can be extracted by SFA directly without previous resolution of their concentration profiles. The resolved mass spectrum of component 1 is shown in Figure 4.

**Figure 5:**
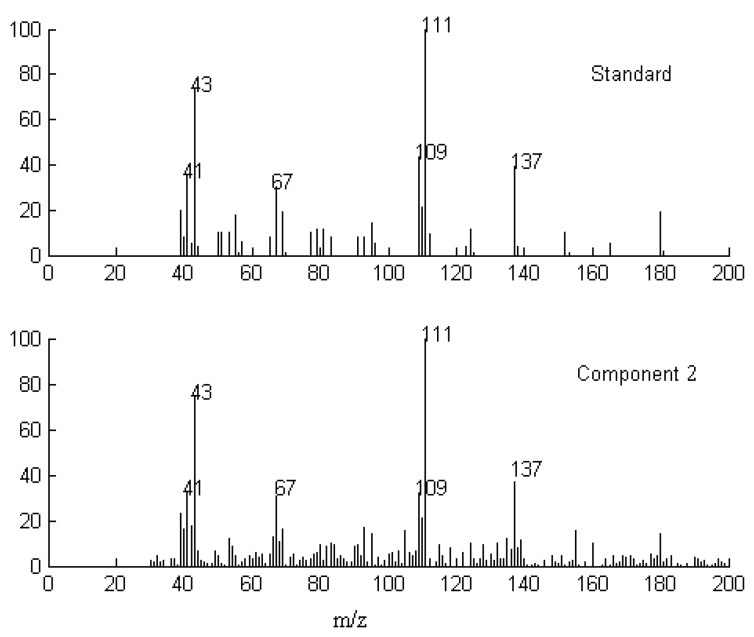
Resolved mass spectrum of component 2 by SFA and standard mass spectrum of dihydroactinidiolide.

**Figure 6:**
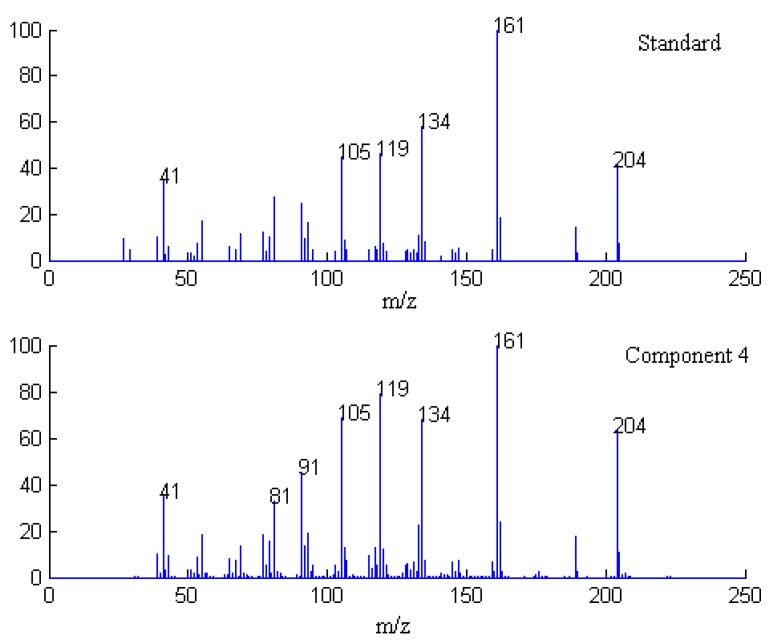
Resolved mass spectrum of component 4 by SFA and standard mass spectrum of δ-cadinene.

**Figure 7:**
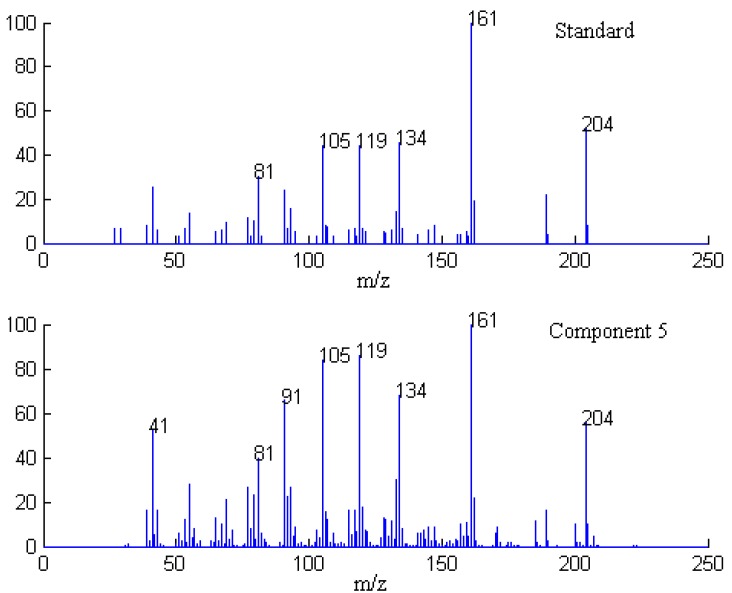
Resolved mass spectrum of component 5 by SFA and standard mass spectrum of β-cadinece.

**Figure 8:**
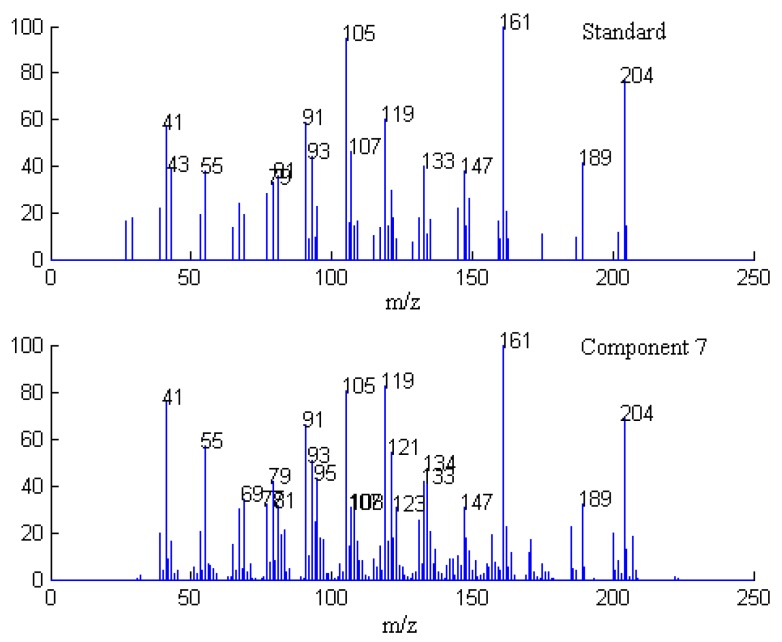
Resolved mass spectrum of component 7 by SFA and standard mass spectrum of valencene.

**Figure 9:**
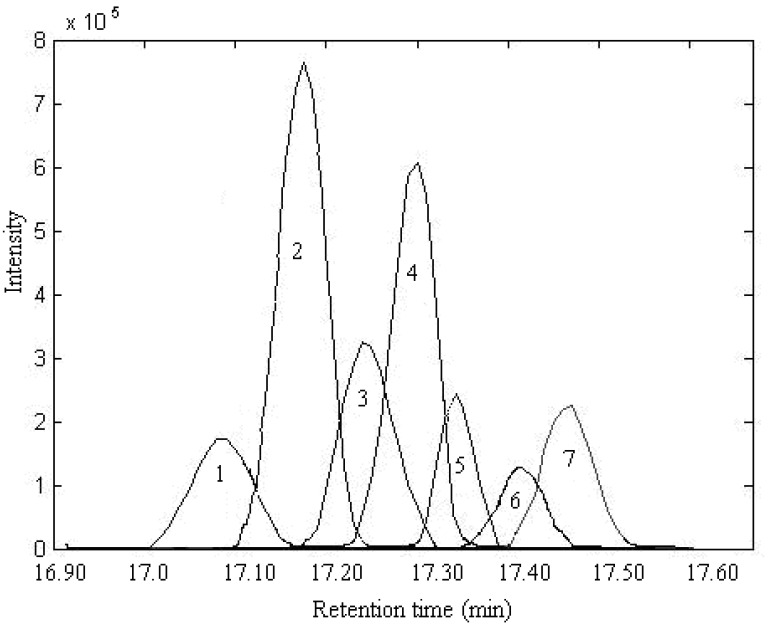
Resolved chromatographic profiles of peak cluster A.

In the same way, the spectra of the other components were obtained, and the corresponding resolved mass spectra of each component are shown in Figure 5, Figure 6, Figure 7, Figure 8. After all the pure spectra were obtained, the concentration profiles could be achieved by using the prior information of the spectra and the linear regression ***C*** = ***XS*** (***S****^T^**S***)^−1^, as shown in Figure 9.

Finally, when each pure spectrum was extracted and the resolved chromatographic profiles of these seven components were obtained, their identification can be done by similarity searches in the NIST mass spectral database. Components 1, 2, 4, 5, and 7 in this cluster may be β-guaiene, dihydroactinidiolide, δ-cadinene, β-cadinece and valencene, based on their similarity matching results of 0.93, 0.99, 0.97, 0.93, and 0.95, respectively. The accuracy and reliability of the results were thus increased greatly. The corresponding standard spectra of each component in cluster A are shown in Figure 4, Figure 5, Figure 6, Figure 7, Figure 8. Components 3 and 6 were not identified due to their very low match values. Likewise, the spectra of components in other segments can be obtained. The qualitative results are listed in Table 1. Eighty two constituents were resolved, and among them 65 components were identified. Unfortunately, 27 components remain unidentified, either because of the low signal-to-noise ratio or the absence of the compound from the mass spectral database, and some of the component identifications should be viewed as tentative.

**Table 1:** Identification and quantification of compounds in the essential oil from Flemingiae Macrophyllae and Radix Flemingiae Latifolia Benth.

Series No.	Retention time (min)		Compound name	Molecularformula	Relative content (%)
	*X*_1_^a^	*X*_2_^b^			
1	3.439	-	Prenal	C_5_H_8_O	0.07
2	3.653	3.658	Hexanal	C_6_H_12_O	0.35
3	6.368	6.372	2-Amylfuran	C_9_H_14_O	0.03
4	7.065	-	D-Limonene	C_9_H_20_	0.27
5	7.979	7.984	Linalool	C_10_H_18_O	0.98
6	8.682	8.691	L-Camphor	C_10_H_16_O	1.91
7	9.104	-	l-2-Bornanol	C_10_H_18_O	0.06
8	9.267	9.275	*p*-Menth-1-en-4-ol	C_10_H_18_O	0.66
9	9.419	9.428	*p*-Menth-1-en-8-ol	C_10_H_18_O	0.87
10	10.529	10.536	Nonanoic acid	C_9_H_18_O_2_	0.46
11	10.671	-	Perillyl aldehyde	C_10_H_14_O	0.04
12	10.914	10.925	Anethole	C_10_H_12_O	1.62
13	11.065	11.081	Bornyl acetate	C_12_H_20_O_2_	0.72
14	11.294	11.308	Tricyclo[3.2.1.02,7]oct-3-ene, 2,3,4,5-tetramethyl-	C_12_H_ 18_	0.57
15	11.504	-	Furan, 2,5-dibutyl-	C_12_H_20_O	0.03
16	12.465	12.478	Decanoic acid	C_10_H_20_O_2_	0.06
17	12.720	12.731	α-Cubebene	C_15_H_24_	0.56
18	12.859	12.875	α-Longipinene	C_15_H_24_	1.47
19	13.325	13.331	Ylangene	C_15_H_24_	1.69
20	13.476	13.493	Longicyclene	C_15_H_24_	2.84
21	13.771	-	Copaene	C_15_H_24_	0.64
22	14.305	14.312	Longofolene	C_15_H_24_	3.21
23	14.437	-	Di-*epi*-α-cedrene	C_15_H_26_	0.08
24	14530	14.542	β-Caryophyllene	C_15_H_24_	0.82
25	14.575	14.583	β-Cedrene	C_15_H_24_	5.92
26	14.744	14.762	Germacrene D	C_15_H_24_	0.65
27	15.057	15.071	(*Z*)-β-Farnesene	C_15_H_24_	0.61
28	15.108	-	Himachala-2,4-diene	C_15_H_24_	0.07
29	15.385	15.435	α-Himachalene	C_15_H_24_	4.13
30	15.572	15.590	α-Caryophyllene	C_15_H_24_	0.44
31	15.593	-	Acoradiene	C_15_H_24_	0.33
32	15.673	15.685	Dihydrocurcumene	C_15_H_24_	0.03
33	15.858	15.869	Eremophilene	C_15_H_24_	1.30
34	16.006	16.021	(+)-Cycloisosativene	C_15_H_24_	0.05
35	16.108	16.117	Humulen-(v1)	C_15_H_24_	0.92
36	16.210	16.215	Longifolene-(V4)	C_15_H_24_	6.82
37	16.321	16.327	α-Guaiene	C_15_H_24_	0.49
38	16.419	-	Patchoulene	C_15_H_24_	0.88
39	16.721	16.730	β-Himachalene	C_15_H_24_	5.26
40	17.077	17.086	β-Guaiene	C_15_H_24_	0.72
41	17.162	17.184	Dihydroactinidiolide	C_11_H_16_O_2_	2.14
42	17.275	17.292	δ-Cadinene	C_15_H_24_	1.53
43	17.321	17.334	β-Cadinece	C_15_H_24_	0.56
44	17.510	-	Valencene	C_15_H_24_	0.67
45	18.710	18.722	Caryophyllenyl alcohol	C_15_H_26_O	0.31
46	19.076	19.085	Caryophyllene oxide	C_15_H_24_O	0.85
47	19.142	19.155	Drimenol	C_15_H_26_O	1.27
48	19.470	-	3-Isobutyl-4,5-dimethyl-3*H*-isobenzofuran-1-one	C_14_H_18_O_2_	0.06
49	19.732	19.740	Cedrol	C_15_H_26_O	0.83
50	19.918	19.932	Bulnesol	C_15_H_26_O	4.17
51	20.235	-	Mansonone C	C15H_26_O_2_	0.07
52	20.342	20.360	Epiglobulol	C15H_26_O	0.78
53	20.621	20.647	Cubenol	C_15_H_26_O	1.74
54	20.967	20.982	ι-Cadinol	C_15_H_26_O	0.56
55	21.151	21.165	δ-Cadinol	C_15_H_26_O	1.82
56	21.494	21.522	Torreyol	C_15_H_26_O	0.38
57	21.662	21.684	β-Selinenol	C_15_H_26_O	7.12
58	21.867	21.887	α-Eudesmol	C15H26O	5.16
59	22.061	22.077	Cadalene	C_15_H_18_	2.22
60	23.167	23.185	Hedycaryol	C_15_H_26_O	0.51
61	23.286	23.310	Peruviol	C_15_H_26_O	1.26
62	26.172	26.190	Farnesol isomer a	C_15_H_26_O	4.87
63	26.362	26.384	*cis*-Farnesal	C_15_H_24_O	0.46
64	26.471	-	Tetradecanoic acid	C_14_H_28_O_2_	0.24
65	33.571	33.590	Hexadecanoic acid	C_16_H_32_O_2_	0.58

^a^ Representing the sample from Flemingiae Macrophyllae. ^b^ Representing the sample from Flemingiae Latifolia Benth. - Correlative component is not found in ***X***_2_.

### 2.2. Quantitative Analysis

After the pure chromatographic profile and mass spectrum of each component were resolved, the total two-way response of each component can be obtained from the outer product of the concentration vector and the spectrum vector for each component, namely **C*_i_*S*_i_****^T^*. Similar to the general chromatographic quantitative method with peak area or height, the concentration of each component is proportional to the overall volume of its two-way response (**C*_i_*S*_i_****^T^*). The final relative quantitative results were also listed in Table 1. The 65 components which have been identified in Radix Flemingiae Macrophyllae accounted for 88.79% of the volatile components. 

### 2.3. Identification of Common Components

**Figure 10:**
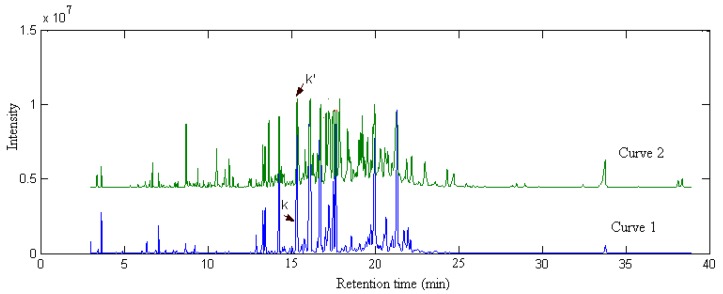
The total ionic current chromatograms of the volatile components in Radix Flemingiae Macrophyllae (***X***_1_: Curve1) and of the volatile components in Radix Flemingiae Latifolia Benth (***X***_2_: Curve 2) obtained from GC-MS, respectively. *k* is target component in Radix Flemingiae Macrophyllae and and *k’* is the correlative component in Flemingiae Latifolia Benth.

Curve 1 and curve 2 in Figure 10 are the TIC chromatograms of response ***X***_1_ from Radix Flemingiae Macrophyllae and ***X***_2_ from Radix Flemingiae Latifolia Benth obtained from GC-MS, respectively. It can be seen from Figure 10 that ***X***_2_ was consistent in eluting components with ***X***_1_, but the concentration distribution of some individual components were different. To compare and distinguish Radix Flemingiae Macrophyllae and its substitutes, such as Radix Flemingiae Latifolia Benth, one may extract pure component spectra from response ***X***_2_ and identify them once again. However, the *a priori* information of ***X***_1_ may be used to reduce some unnecessary work when we compare the quality of Radix Flemingiae Macrophyllae and its substitutes, such as Radix Flemingiae Latifolia Benth. Here, spectral correlative chromatography (SCC) [16] was used to identify each common component directly from the known information of ***X***_1_ instead of resolving each sample data one by one.

A brief depiction of spectral correlative chromatography was given as follows. Generally one substance has only a spectrum, no matter whether it exists in different samples, of course, it is best if they are comparable experiments. Suppose that ***X***_1_ and ***X***_2_ are two response matrices from two different samples of Chinese herbs, where ***s****_i_* is the spectrum of the *i*th component of ***X***_1_ and ***s****_j_* the spectrum of the *j*th component of ***X***_2_, respectively. The correlation coefficient of these two components can be written as the product of two spectral vectors as follows:

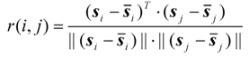
(1)
where 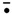 denotes the mean of the spectral vector, and 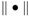 is Frobenius norm. The values *r*(*i, j*) of spectral correlation coefficient are in the range −1 ≤ *r* ≤ 1, and the larger the value of *r* is the more correlative are the components between the *i*th and the *j*th peak. When *r* equals to 1, these two components are identical. On account of errors and interference from noise and background, *etc.,* in actual systems, the maximum of *r* is not equal to 1 but close to 1. In order to check if the component existing in ***X***_1_ really also exists in ***X***_2_, we may first extract ***s****_i_* (the component to be investigated) from ***X***_1_ and then calculate its corresponding correlation coefficient with every row vector, say ***x****_j_**^T^*** (*j* = 1, …, *m*), in ***X***_2_ as shown in Equation (2)?:


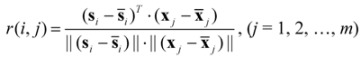
(2)

Accordingly, a spectral correlation coefficient curve is obtained by calculating *r*(*i, j*) of the spectral vector ***s****_i_* against every row of ***X***_2_ in sequence. The curve with the same length of the eluting chromatogram contains therefore the spectral correlation information in ***X***_2_ corresponding to the component spectrum, say ***s****_i_*, in ***X***_1_. With the help of the correlation coefficient curve thus obtained, one may easily pick up the correlation information between ***X***_1_ and ***X***_2_. Consequently, the curve was named spectral correlative chromatography (SCC) in this work.

The detailed procedure is illustrated by an example, pure component k (α-himachalene) of ***X***_1_. Its spectrum has been extracted with the SFA method. How to extract the common components from ***X***_2_ with the SCC method?

**Figure 11:**
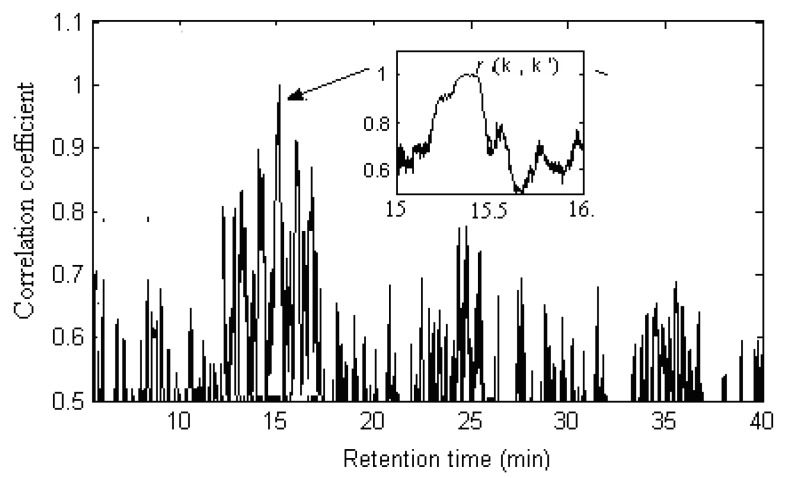
Correlative chromatogram of component *k* of sample ***X***_1_ against those of ***X***_2_. Right top part is the amplified segment where component k′ from Flemingiae Latifolia Benth exists and r(*k*, *k′*) is the correlation coefficient of component *k* and *k′*.

A series of spectral correlation coefficients (*r*) were obtained by calculating the spectrum ***s****_k _*of component k against every row of ***X***_2_ in sequence (see Figure 11) according to Equation (2). As shown in Figure 11, the correlation coefficients *r*(k, k’) corresponding to components k’ of ***X***_2_ were larger than the others. As shown in middle top part of Figure 11, the value of *r*(k, k’) is 0.9981. If the errors and interference from noise and background, *etc.* in actual systems was taken into account, the correlation coefficient was quite close to 1. Then it was concluded that components k’ and k were correlative based on spectral-dependent principle of substance identification. In this way, other correlative components in ***X***_2_ could be obtained. There were 51 common components between Radix Flemingiae Macrophyllae and Flemingiae Latifolia Benth. The results were also listed in Table 1.

### 2.4. Comparison of Samples

The procedure performed for Flemingiae Macrophyllae was used for those peaks in Flemingiae Latifolia Benth, which were not identified by the SCC method. Thirty components in the essential oil of Flemingiae Latifolia Benth, which were not found in Flemingiae Macrophyllae, were identified with the SFA method. The results are listed in Table 2. Among the 79 separated constituents in the essential oil of Flemingiae Latifolia Benth, a total of 64 components were identified. Fifty one identified components existed in both Flemingiae Macrophyllae and Flemingiae Latifolia Benth. Alkenes, such as longifolene-V4, β-cedrene and β-himachalene, and alcohols, such as β-selinenol, α-eudesmol and farnesol isomer, were the main volatile constituents in both Flemingiae Macrophyllae and Flemingiae Latifolia Benth, but the contents of each component were different. The obtained results can provide useful information for further development of Flemingiae Macrophyllae.

**Table 2:** Qualitative results of some other constituents in the essential oil from Flemingiae Latifolia Benth.

Series No.	Retention time (min)	Compound name	Molecule structure
1	6.914	*m*-Cymene	C_10_H_16_
2	9.128	Octanoic acid	C_8_H_16_O_2_
3	10.719	4-Hydroxy-3-methylacetophenone	C_9_H_10_O_2_
4	11.526	Di-epi-α-cedrene	C_10_H_18_O
5	14.430	α-Cedrene	C_15_H_32_
6	15.112	1 *H*-Benzocycloheptene, 2,4a,5,6,7,8,9,9a-octahydro-3,5,5-trimethyl-9-methylene-	C_15_H_24_
7	15.672	Dihydrocurcumene	C_15_H_24_
8	16.541	α-Muurolene	C_15_H_24_
9	17.512	Eudesma-3,7(11)-diene	C_15_H_24_
10	19.513	Caryophyllenyl alcohol	C_15_H_26_O
11	20.247	1-Cyclohexen-1-ol, 2,6-dimethyl-, acetate	C_10_H_16_O_2_
12	26.490	(*E*)-10-Pentadecenol	C_15_H_30_O
13	34.172	*cis*-7-Tetradecen-1-ol	C_14_H_28_O

## 3. Experimental

### 3.1. Instrumentation and Materials

Radix Flemingiae Macrophyllae was purchased from a Zhejiang pharmaceutical store and was identified by a researcher from the Institute of Materia Medica, Hunan Academy of Traditional Chinese Medicine and Materia Medica. Radix Flemingiae Latifolia Benth, a substitute of Radix Flemingiae Macrophyllae was also purchased from a local pharmaceutical store in Zhejiang Province. Anhydrous sodium sulfate and ether were purchased from Shanghai Chemical Reagent Research Institute (Shanghai, P.R. China) and were of analytical grade. GC-MS was performed with a Shimadzu GC-2010 gas chromatography instrument coupled with a Shimadzu 2010 mass spectrometer. The volatile constituents in Radix Flemingiae Macrophyllae and Radix Flemingiae Latifolia Benth were separated on a 30 m × 0.25 mm I.D. fused silica capillary column coated with 0.25 µm OV-1 film.

### 3.2. Extraction of the Essential Oil

The samples were dried at constant temperature (40 °C) for 2 h. Then, 500 g distilled water and 200 g Radix Flemingiae Macrophyllae or Radix Flemingiae Latifolia Benth were added into a standard apparatus, and the volatile constituents were extracted by water distillation for 8 h according to the procedure described in the Chinese Pharmacopoeia [19]. Effluent was extracted with diethyl ether and the diethyl ether was removed under low temperature. The obtained essential oils were dried with anhydrous sodium sulfate and stored in the refrigerator at 4 °C prior to analysis.

### 3.3. Gas Chromatography-Mass Spectrometry

Analytical conditions were as follows: the oven was held at 50 °C for 1 min during injection, then programmed at 10 °C·min^−1^ to a temperature of 150 °C, finally ramped at 2 °C·min^−1^ to a temperature of 200 °C and held for 5 min. Inlet temperature was kept at 260°C all the time. A 1.0 µL volume of essential oil was injected into the GC. Helium carrier gas at a constant flow-rate of 1.0 mL·min^−1^ and a 10:1 split ratio were used simultaneously. Mass spectrometer was operated in full scan and electron impact (EI+) modes with an electron energy of 70 eV. Interface temperature was 250 °C. MS source temperature was 200 °C. In the range of *m/z* 30 to 350, mass spectra were recorded with 0.2 s·scan^−1^ velocity.

### 3.4. Data Analysis

Data analysis was performed on a Pentium based IBM compatible personal computer. All programs for the chemometrical resolution methods were coded in MATLAB 6.5 for Windows. The library searches and spectral matching of the resolved pure components were conducted on the National Institute of Standards and Technology (NIST) MS database containing about 107,000 compounds.

## 4. Conclusions

In this work, a combined chemometrics method, subwindow factor analysis (SFA) and spectral correlative chromatography (SCC), were used for analysis of the volatile constituents in Radix Flemingiae Macrophyllae and Radix Flemingiae Latifolia Benth, one of its common substitutes. This study showed that the application of this combined approach is a powerful tool, which comprehensively reveals the quality and quantity of chemical constituents of traditional medicines for effective evaluation of similarity or differences between complex analytical samples.
